# The fungal phytotoxin lasiojasmonate A activates the plant jasmonic acid pathway

**DOI:** 10.1093/jxb/ery114

**Published:** 2018-03-24

**Authors:** Andrea Chini, Alessio Cimmino, Marco Masi, Pierluigi Reveglia, Paola Nocera, Roberto Solano, Antonio Evidente

**Affiliations:** 1 Department of Plant Molecular Genetics, Centro Nacional de Biotecnología, Consejo Superior de Investigaciones Científicas, Madrid, Spain; 2 Dipartimento di Scienze Chimiche, Università di Napoli Federico II, Complesso Universitario Monte Sant’Angelo, Napoli, Italy

**Keywords:** Jasmonate signalling pathway, jasmonic acid, *Lasiodiplodia*, lasiojasmonate A, phytotoxins

## Abstract

Jasmonates are signaling compounds that regulate plant responses to stress. Jasmonic acid (JA) is the direct precursor of the bioactive plant hormone JA-isoleucine (JA-Ile), the ligand of the CORONATINE INSENSITIVE 1–jasmonate ZIM-domain (COI1–JAZ) co-receptor complex. JA, its methyl ester, and three furanonyl esters were recently isolated from the grapevine pathogen *Lasiodiplodia mediterranea*. The JA ester lasiojasmonate A (LasA) is the first reported naturally occurring JA-furanone, and its mode of action has not yet been elucidated. Here, we show that LasA activates many JA-regulated responses *in planta*, including protein degradation, gene expression, and physiological processes. These *in vivo* effects require LasA conversion into JA, formation of JA-Ile, and its recognition by the plant JA-Ile perception complex. These findings suggest a mode of action of the natural fungal LasA as an inactive JA pool that can be transformed into the bioactive JA-Ile form. We propose that fungal production of JA derivates such as LasA occurs at late infection stages to induce plant JA responses such as cell death, and can facilitate fungal infection.

## Teaser

The fungal jasmonate lasiojasmonate A functions as a source of jasmonic acid that activates the JA-isoleucine pathway in Arabidopsis.

## Introduction

Fungi are producers of secondary metabolites that belong to different classes of natural compounds (Turner and Aldridge 1983; Cole *et al.*, 2003; Lo Presti *et al.*, 2015; Fonseca *et al.*, 2018). Phytopathogenic fungi are among the main causative agents of disease in crops and forest plants, and lead to considerable economic losses (Evidente and Motta 2001; Evidente *et al.*, 2010, 2011, 2013; Cimmino *et al.*, 2014). For example, Botryosphaeriaceae is a widely spread family of pathogenic plant fungi associated with fruit rot, leaf spots, dieback, cankers, and root rot of angiosperms and gymnosperms (Phillips *et al.*, 2013). Several species of the Botryosphaeriaceae, including *Lasiodiplodia*, infect grapevine worldwide (Larignon *et al.*, 2001; Phillips, 2002; Van Niekerk *et al.*, 2006; Pitt *et al.*, 2010; Úrbez-Torres, 2011). *In vitro*, the newly discovered species *Lasiodiplodia mediterranea* (Linaldeddu *et al.*, 2015) produces (1*R*,2*R*)-(-)-jasmonic acid (JA) as its main phytotoxin, the methyl ester (JA-Me), and three furanonenoyl esters, termed lasiojasmonates A, B, and C (LasA, B, C), as well as 16-*O*-acetylbotryosphaerilactones A and C, botryosphaerilactone A, (3*S*,4*R*,5*R*)-4-hydroxymethyl-3,5-dimethyldihydro-2-furanone, and (3*R*,4*S*)-botryodiplodin (Andolfi *et al.*, 2014).

Jasmonic acid (JA) belongs to the naturally occurring family of compounds termed jasmonates (JAs); these are key plant signalling molecules that regulate several stress responses as well as developmental traits (Wasternack and Hause, 2013; Chini *et al.*, 2016; Wasternack and Feussner, 2018). JA activates plant defenses against nematodes, herbivores, and necrotrophic pathogens (Wasternack and Hause, 2013; Goossens *et al.*, 2016; Guo *et al.*, 2018). JAs are cyclopentanone oxylipins derived from α-linolenic acid via lipid peroxidation (Schaller and Stintzi, 2009). The JA biosynthetic pathway and its intermediate compounds, such as 12-oxo-phytodienoic acid (OPDA), have been analysed extensively in plants (Schaller and Stintzi, 2009; Wasternack and Hause, 2013; Wasternack and Feussner, 2018; Wasternack and Song, 2017; Chini *et al.*, 2018). In the final biosynthetic step of this pathway, the JA-amido synthetase JAR1 conjugates JA with isoleucine (Ile) to generate bioactive JA-Ile [(+)-7-*iso*-JA-Ile], which is in equilibrium with its inactive epimer (-)-JA-Ile (Staswick and Tiryaki, 2004; Fonseca *et al.*, 2009). Bioactive JA-Ile acts as ‘molecular glue’ to promote formation of the COI1-JAZ (CORONATINE INSENSITIVE 1–jasmonate ZIM-domain) co-receptor complex (Chini *et al.*, 2007; Thines *et al.*, 2007; Katsir *et al.*, 2008; Fonseca *et al.*, 2009; Sheard *et al.*, 2010). JA-Ile-induced COI1–JAZ interaction promotes ubiquitination and proteasome degradation of the JAZ repressors, which in turn activate several transcription factors that regulate specific physiological responses (Chini *et al.*, 2007, 2016; Thines *et al.*, 2007; Zhang *et al.*, 2015; Wasternack and Feussner, 2018; Zhai *et al.*, 2017).

Synthesis of phytohormones and phytohormone mimics is a common strategy of several pathogens to unbalance the plant immune system (Gimenez-Ibanez *et al.*, 2016). The most-studied example is probably coronatine (COR), which is produced by some strains of *Pseudomonas syringae*. COR mimics the bioactive JA-Ile hormone by directly binding to the JA-Ile receptor COI1–JAZ (Brooks *et al*., 2005; Sheard *et al.*, 2010). *Lasiodiplodia mediterranea* and other fungi also accumulate oxylipins such as OPDA, JA, and JA conjugates (Miersch *et al.*, 1993; Andolfi *et al.*, 2014; Chanclud and Morel, 2016; Fonseca *et al.*, 2018). For example, *Botryodiplodia theobromae*, the causal agent of dieback disease of several plants, produces JA as a phytotoxin (Miersch *et al.*, 1987; Gimenez-Ibanez *et al.*, 2016). *Fusarium oxysporum* not only produces JA, but some strains also accumulate jasmonate conjugates such as JA-Ile and JA-Leu (Miersch *et al.*, 1999; Brodhun *et al.*, 2013; Cole *et al.*, 2014). To promote infection, the activity of these jasmonates from *F. oxysporum* requires COI1, the plant JA-Ile receptor; this suggests a role for fungal JA-conjugates as virulent effectors (Cole *et al.*, 2014). The rice blast fungus *Magnaporthe oryzae* was recently shown to convert natural JA into hydroxylated JA (12OH-JA), an inactive product of JA catabolism (Patkar *et al.*, 2015). Although the activity of fungus-produced 12OH-JA is still not clear, secretion of this hydroxylated compound during infection to weaken plant defences suggests a role as a virulent factor (Patkar *et al.*, 2015).

Although several JA derivatives have been isolated as fungal metabolites, their mode of action has not yet been determined. Here, we studied the effect of the natural fungal JA derivative LasA on plant JA-regulated responses, to define its mode of action *in planta*. LasA triggers many JA-regulated plant responses, including JAZ degradation, activation of JA gene expression, and growth inhibition. These effects *in planta* are dependent on *JAR1* and *COI1*, which shows that plant conversion of LasA into JA, generation of JA-Ile, and its recognition by the JA-Ile receptor complex are necessary for the fungal LasA activity.

## Materials and methods

### Chemicals

Jasmonic acid and (1*R*,2*R*,3*S*,4*R*,5*R*)-4-hydroxymethyl-3,5-dimethyldihydro-2-furanone (LasA) were isolated from culture filtrates of *Lasiodiplodia mediterranea* (BL 101 strain), as reported by Andolfi *et al.* (2014). Coronatine was purchased from Sigma-Aldrich.

### Plant material and growth conditions

*Arabidopsis thaliana* Col-0 was the genetic background of wild-type and mutant lines used in this study. Knock-out lines for *coi1-1* (Xie *et al.*, 1998), *coi1-30* (Yang *et al.*, 2012), *coi1-2* (Xu *et al.*, 2002), *jar1-1* (Staswick and Tiryaki, 2004), *myc2/jin1-2* (Lorenzo *et al.*, 2004), and *jai3-1* (Chini *et al.*, 2007) have been described previously. Seeds were surface-sterilized by the chlorine gas method (in the presence of bleach and HCl for 3 h) and stratified for 2–3 d at 4 °C in the dark. All of the seedlings were grown under a 16-h light/8-h dark cycle at 21 °C (photosynthetic photon flux density approximately 100 µmol m^–2^ s^–1^). For root-growth inhibition assays, 10 to 30 seeds of each line were germinated for 10 d, with or without the presence of 50 µM jasmonic acid, 50 µM LasA, or 0.5 µM COR (Reveglia *et al.*, 2018). Images were taken with a Nikon D1-x digital camera and root length was estimated using the ImageJ software (https://imagej.nih.gov/ij/). Data were analysed by one-way ANOVA/Tukey HSD *post hoc* tests. Four independent biological replicates (10–30 seedlings each) were measured for each sample with similar results. Experiments were repeated four times with similar results.

### Anthocyanin measurement

Between 10 to 30 seeds of each line were germinated for 10 d, with or without the presence of 50 µM jasmonic acid, 50 µM LasA, or 0.5 µM COR. Between four to ten seedlings were weighed and pooled for each biological replicate; three independent pools were measured for each line. Seedlings were soaked overnight in 1 ml of hydrochloric acid in aqueous methanol (HCl 0.5 N; methanol 80%, v/v). The reference blank was obtained by adding half of the sample solution to a peroxide reagent (one part 30% hydrogen peroxide in nine parts of methanolic hydrochloric acid, 5:1 v/v, 3N), whereas the other half of the solution was diluted with one volume of methanolic hydrochloric acid (5:1 v/v, 3N). Absorbance was measured at 530 nm, and anthocyanin content was expressed as (A530 sample – A530 blank) per g fresh weight. Data were analysed by one-way ANOVA/Tukey HSD *post hoc* tests. Experiments were repeated twice with similar results.

### Yeast two-hybrid system

All constructs were previously reported by Chini *et al.* (2007). pGAD (prey) and pGBK (bait) plasmids were co-transformed in *Saccharomyces cerevisiae* AH109 cells using standard heat-shock protocols (Chini *et al.*, 2007). Successfully transformed colonies were selected 3 d after transformation on yeast synthetic drop-out medium lacking Leu and Trp (–2 medium). Yeast colonies were then grown in selective –2 liquid medium for ~5 h and cell density adjusted to 3 × 10^7^ cells ml^–1^ (OD_600_=1). Cell suspensions (5 µl) were plated on yeast synthetic drop-out medium lacking Ade, His, Leu, and Trp (–4 medium) to evaluate protein interactions. Where indicated, the medium was supplemented with 20 µM coronatine and/or 100 µM LasA. Plates were incubated (2–6 d; 28 °C) and images were acquired with a Nikon D1-x digital camera.

### JAZ1-GUS staining and quantification assays

The 35S:JAZ1-GUS in the wild-type and *coi1-30* backgrounds have been described previously (Thines *et al.*, 2007; Monte *et al.*, 2014). The 35S:JAZ1-GUS marker line was introgressed into the *jar1-1* background by crossing, and a double-homozygous line was used for further analyses. 35S:JAZ1-GUS seedlings were grown vertically on Murashige and Skoog (MS) plates, whereas 35S:JAZ1-GUS *coi1-30* seedlings were selected vertically on MS plates in the presence of 0.5 µM COR, and 6-d-old seedlings were treated for 1 h with either liquid Johnson medium (control), 5 µM JA, 100 µM LasA, or 1 µM COR solution, as described previously (Chini, 2014; Reveglia *et al.*, 2018). Samples were then placed in standard GUS staining solution [50 mM phosphate buffer, pH 7; 0.5 mM K_4_Fe(CN)_6_; 0.5 mM K_3_Fe(CN)_6_ 0.2% Triton X-100; 0.7 mg ml^–1^ 5-bromo-4-chloro-3-indolyl β-D-glucuronic acid (X-Gluc)] and incubated overnight at 37 °C (Fernández-Calvo *et al.*, 2011) to visualize GUS activity. Root images were acquired with a Nikon D1-x camera. The analysis was performed using 10–30 plants per sample; the experiment was repeated three times with similar results. For quantification of JAZ1-GUS protein degradation, approximately 50 seedlings were treated as described above, and the roots were collected and frozen in liquid nitrogen as previously described (Monte *et al.*, 2014). The roots were homogenized in extraction buffer containing 50 mM phosphate buffer, pH 7, 10 µM β-mercaptoethanol, 10 mM EDTA, 0.1% sarcosyl (*N*-lauroylsarcosine sodium salt), and 0.1% Triton X-100. The total protein content was quantified by the Bradford method (Bio Rad Protein Assay). Then, a 30-μl extract was incubated with 70 μl protein extraction buffer containing 1 mM MUG (methylumbelliferyl-β-D-glucuronide hydrate) for 1 h at 37 °C, and 10-μl samples were taken at *t*=0 and *t*=1 h. Finally, 90 μl of 0.2 M Na_2_CO_3_ was used to stop the reaction. Fluorescence was measured at 365/460 nm (excitation/emission) with a SpectraMax M2 spectrophotometer (Molecular Devices). Six independent replicates were measured.

### pJAZ2-GUS expression assays

The *pJAZ2:GUS* marker line was described previously by Gimenez-Ibanez *et al.* (2017). For *pJAZ2:GUS* expression experiments, seeds were germinated as described above and 6-d-old seedlings were treated with either liquid Johnson medium (control), 5 µM JA, or 100 µM LasA (2 h); GUS was stained as described above and incubated overnight at 37 ºC. Images were acquired with a Nikon D1-x camera. Analyses were performed using 10–20 plants per sample and the experiment was repeated three times with similar results.

### Quantitative RT-PCR

Quantitative RT-PCR was performed using biological samples of tissue pooled from 10–20 wild-type seedlings. RNA was extracted and purified using Trizol reagent (Invitrogen) followed by use of a High Pure RNA isolation kit (Roche), including DNase digestion to remove genomic DNA contamination. cDNA was synthesized from 1 μg total RNA with a high-capacity cDNA reverse transcription kit (Applied Biosystems). For gene amplification, 4 μl from a 1:10 cDNA dilution was added to 4 μl of EvaGreen® qPCR Mix Plus (Solis BioDyne) and gene-specific primers as previously described (Chini *et al.*, 2018). Quantitative PCR was performed in 96-well optical plates in a HT 7900 Real Time PCR system (Applied Biosystems) using standard thermocycler conditions (an initial hold at 95 °C for 10 min, followed by a two-step SYBRPCR program of 95 °C for 15 s and 60 °C for 60 s for 40 cycles). Relative expression values given as the means of three or four technical replicates relative to the basal wild-type control using *ACT8* as the housekeeping gene. Data were analysed using unpaired Student’s *t*-tests.

## Results

### Effect of LasA *in planta*

**Fig. 1.**
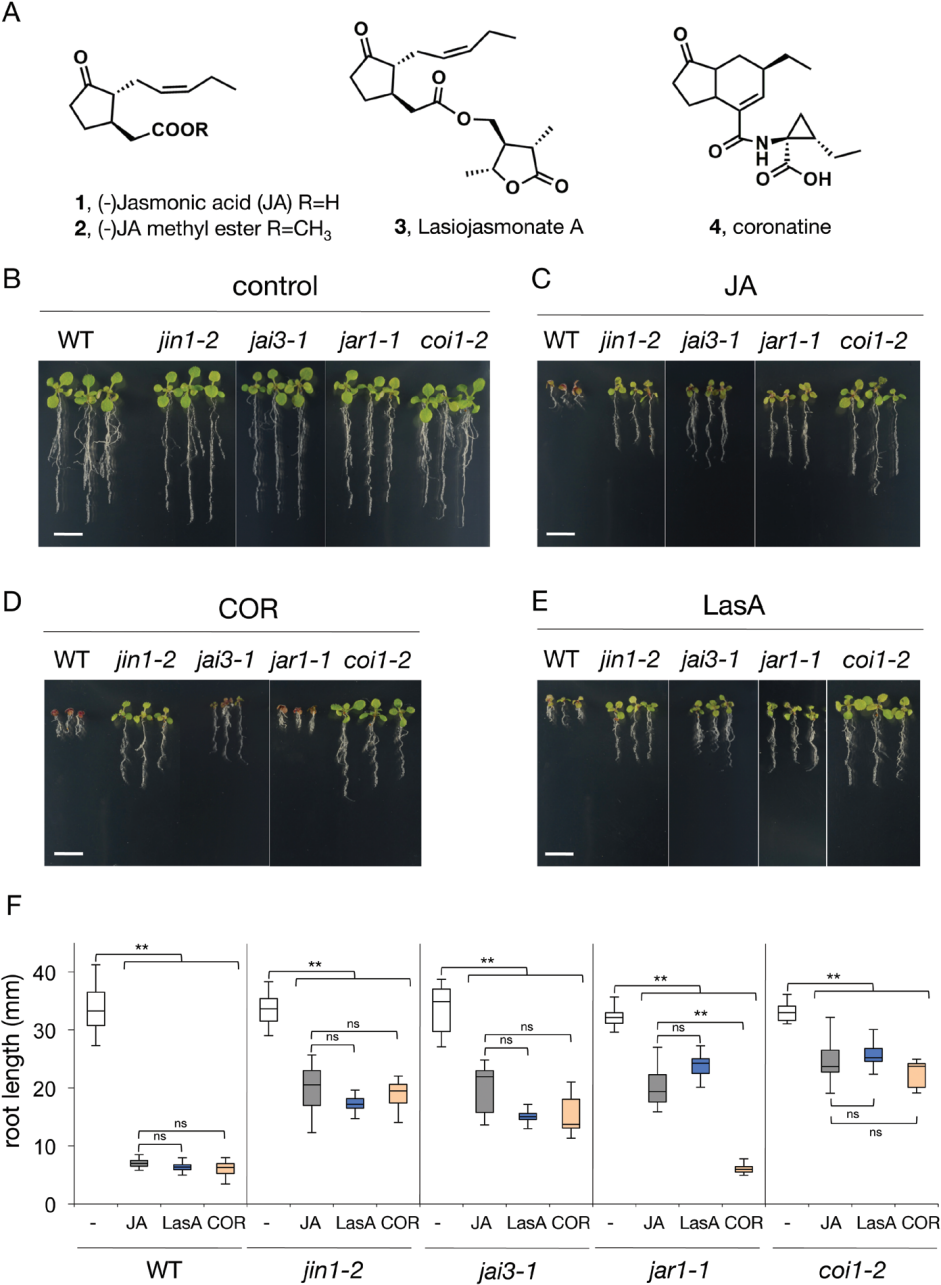
Effects of LasA on Arabidopsis plants. (A) Structure of jasmonic acid (**1**), methyl jasmonate (**2**), lasiojasmonate A (**3**), and COR (**4**). (B–F) Wild-type (Col-0) and mutant seedlings (*n*=8–25) were germinated in the absence (control, B) or presence of 50 µM JA (C), 50 µM LasA (D), or 0.5 µM COR (E). Scale bars are 1 cm. (F) Root length was measured 10 d after germination. Data are shown as box-plots (control in white boxes, treatments with JA, LasA, and COR in grey, blue, and orange, respectively); horizontal lines are medians, the boxes show the upper and lower quartiles, and the whiskers show the full data range. Asterisks indicate significant differences compared to the untreated control or JA treatment for each plant genotype, evaluated by one-way ANOVA/Tukey HSD *post hoc* tests (** *P*<0.01). Experiments were repeated four times with similar results.

(–)-JA, its methyl ester, and lasiojasmonate A (Fig. 1A, 1–3) were purified from the organic extracts of *Lasidiplodia mediterranea* (BL 101 strain) culture filtrates and identified by comparing their physical and spectroscopic properties with those previously reported (Andolfi *et al.*, 2014). To assess its effects *in planta*, wild-type (WT) Arabidopsis plants were grown with or without fungal LasA. Similar to JA, LasA induced growth inhibition and anthocyanin accumulation (Fig. 1B–F; Supplementary Fig. S1 at *JXB* online). To test whether the activity of this JA derivative required *in planta* conjugation of JA to Ile, JA-Ile perception, and the JA signalling pathway, we also grew seeds of *jar1-1* (impaired in JA-Ile conjugation), *coi1-2* (weak allele of the JA-Ile receptor), and *jai3-1* and *jin1-2* (both JA signalling mutants) in the presence of LasA. LasA showed an effect similar to that of JA in all genotypes, strongly inhibiting growth of WT plants but only partially inhibiting *jar1-1*, *jai3-1*, *jin1-2*, and *coi1-2* (Fig. 1B–F; Supplementary Table S1). LasA induced accumulation of the defence secondary metabolites anthocyanins, and this response required a functional JA pathway (Supplementary Fig. S1).

These results show that fungal LasA triggers JA-regulated responses, and that this LasA-induced effect requires the canonical JA pathway.

### Effect of LasA on JA-mediated plant transcriptional activation

**Fig. 2.**
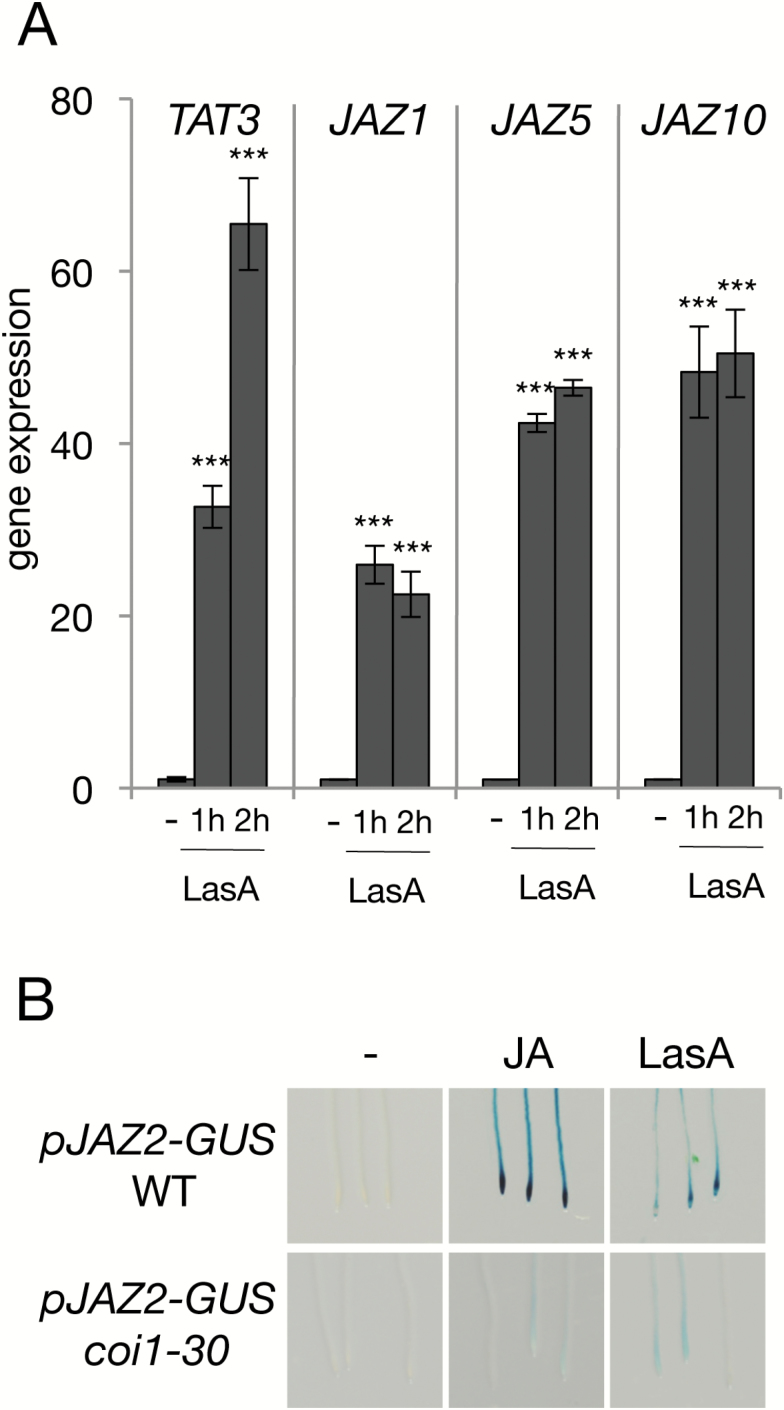
Effects of LasA on JA-regulated transcriptional activation. (A) Gene expression analysis of *TAT3*, *JAZ1*, *JAZ5*, and *JAZ10* in wild-type (Col-0) plants in response to 50 µM LasA for 1 or 2 h; untreated plants (–) were included as controls. *ACT8* was used as the housekeeping control gene. Statistically significant differences in gene expression between controls and LasA-treated plants were determined by two-tailed Student’s *t*-test (*** *P*<0.001). Each biological sample consisted of tissue pooled from 10–15 plants. Data are means (±SD) of three or four technical replicates. (B) GUS-staining for visualization of *pJAZ2-GUS* expression in roots of transgenic Arabidopsis in the wild-type and *coi1-30* backgrounds. Seedlings (7-d-old) were treated with 5 µM JA or 100 µM LasA; untreated plants (–) were included as controls.

As LasA triggers physiological responses mediated by JA, we evaluated whether it could activate JA-dependent gene expression. We analysed the transcriptional changes of well-known JA-marker genes induced by LasA, including several *JAZ* genes as well as JA-biosynthetic genes such as *LOX3*, *AOS*, *AOC1*, and *OPR3*. Exogenous treatment of LasA triggered the expression of all tested JA-marker genes within 1 h (Fig. 2A; Supplementary Fig. S2).

To provide further evidence of the role of LasA in the activation of JA signalling, we assessed the effect of LasA on the JA-inducible marker line *pJAZ2:GUS*. As expected, LasA activated expression of the marker line, although at a lower level than JA (Fig. 2B). To assess whether this activation required JA-Ile perception by COI1, we tested activation of the marker line in the *coi1-30* background. Similar to JA, fungal JA derivatives did not activate *pJAZ2:GUS* expression in *coi1-30* mutant plants (Fig. 2B).

These data indicate that LasA induces expression of JA-regulated genes in a COI1-dependent manner.

### Effect of LasA on the JA-Ile co-receptor COI1–JAZ complex

**Fig. 3.**
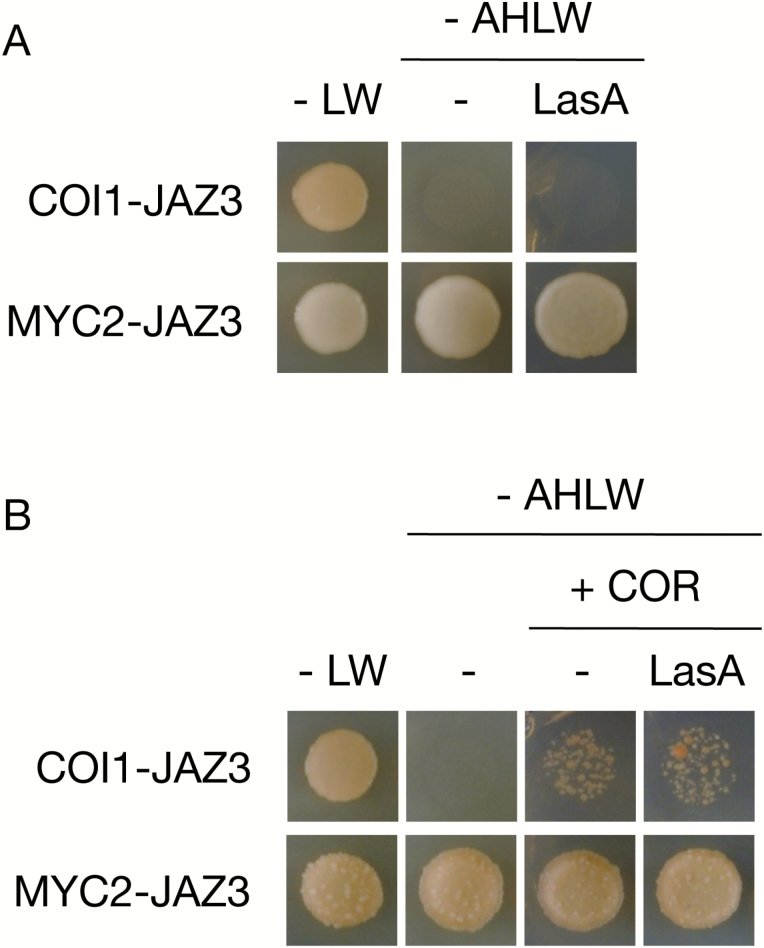
Effects of LasA on the JA-Ile receptor COI1–JAZ. (A, B) Yeast cells co-transformed with pGAD-JAZ3 (prey) and pGBK-COI1 (bait) were selected and subsequently grown on synthetic drop-out medium lacking Leu and Trp (–LW) as the transformation control, or on selective media lacking Ade, His, Leu, and Trp (–AHLW) to test protein interactions. COI1 interaction with JAZ3 was detected only in the presence 20 µM coronatine (B). LasA (at 100 µM) did not alter COI1/JAZ interactions in the presence of COR. As a control, the interaction between JAZ3 (bait) and MYC2 (prey) was also assessed (A, lower panels). LasA did not promote COI1/JAZ3 interaction, nor alter the MYC2/JAZ3 interaction (B, lower panels).

LasA triggers physiological responses mediated by JA in a COI1- and JAR1-dependent manner (Fig. 1), suggesting that it is first converted to JA, then conjugated to form JA-Ile that would in turn trigger JAZ protein degradation. To support this hypothesis, we evaluated the LasA effect on hormone-mediated formation of the COI1–JAZ co-receptor complex in a yeast-two-hybrid assay (Y2H; Chini *et al.*, 2009; Chini, 2014). LasA did not promote COI1–JAZ3 interaction in the absence of the hormone (Fig. 3A). To assess a possible LasA effect on yeast growth and Y2H detection of protein–protein interactions, we also assessed LasA activity on formation of the complex between JAZ3 and the transcription factor MYC2. LasA did not alter the JAZ3–MYC2 interaction. In the presence of COR, COI1 binds to JAZ3; LasA nonetheless did not affect the COR-induced COI1–JAZ3 interaction (Fig. 3B). These findings show that LasA does not directly interfere with the JA-Ile receptor complex COI1–JAZ.

### Effect of LasA on JAZ turnover

**Fig. 4.**
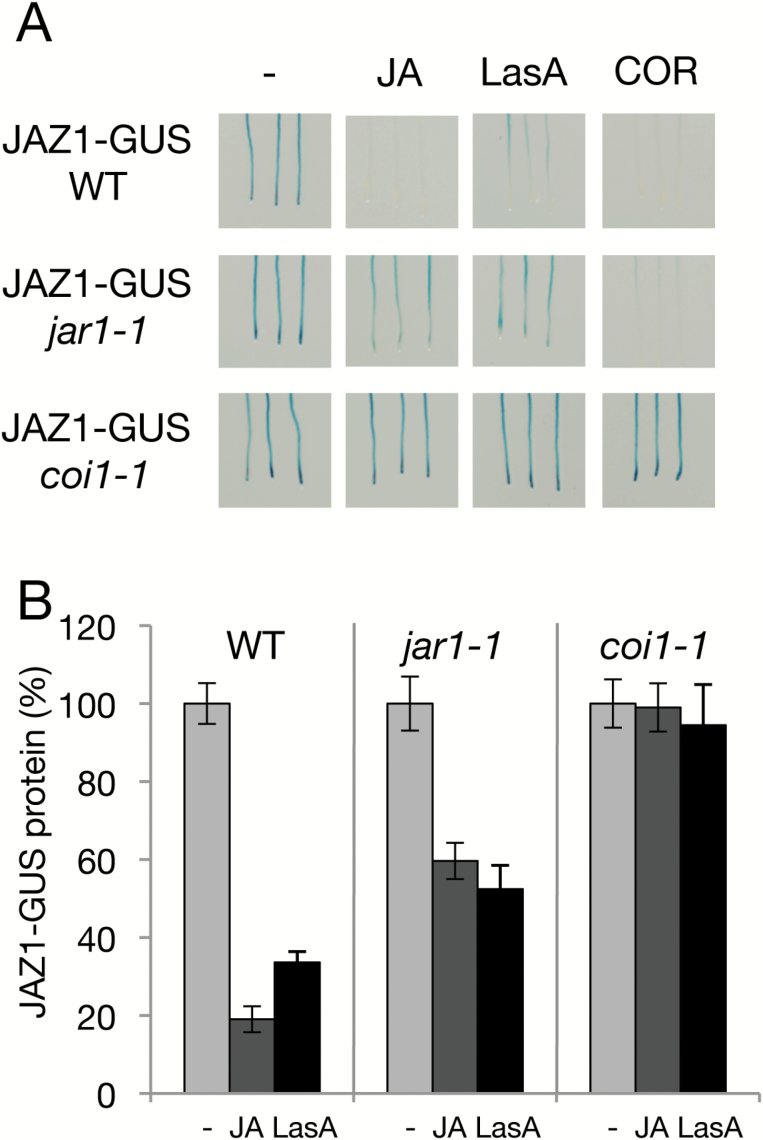
Effects of LasA on JAZ stability. GUS visualization (A) and quantification of GUS activity (B) of JAZ1-GUS in roots of 7-d-old transgenic Arabidopsis 35S:JAZ1-GUS in the wild-type and mutant backgrounds. 35S:JAZ1-GUS wild-type and mutant plants (*n*=20) were treated with 5 µM JA, 100 µM LasA, or 1 µM COR for 1h (A). Untreated control plants (–) are also shown. Experiments were repeated twice with similar results. For JAZ1-GUS quantification, Arabidopsis 35S:JAZ1-GUS in the wild-type and mutant backgrounds (*n*=15–30) were exposed to 25 µM JA or 50 µM LasA for 1 h and GUS activity was measured in the roots (B). Untreated control plants (–) are also shown. Data are the means (±SD) of six replicates.

LasA induced JA-dependent gene expression without interfering directly with the COI1–JAZ receptor; therefore, LasA might be converted to JA and then conjugated to form JA-Ile, which in turn would trigger JAZ protein degradation. To test this hypothesis, we evaluated the effect of LasA on JAZ repressor degradation by studying JAZ1-GUS turnover in the presence of LasA. Similar to JA or COR treatments, LasA induced JAZ1 degradation (Fig. 4A). To determine whether JA must be conjugated into JA-Ile for LasA activity, we assessed the effects of LasA on JAZ1 stability in *jar1-1* mutants, which have partially impaired JA-Ile conjugation. LasA induced a partial JAZ1-GUS degradation, similar to that promoted by JA (Fig. 4A, B). In addition, we studied the effect of LasA in *coi1-1* plants that have loss of JA-Ile receptor function, in order to verify the requirement for JA-Ile perception for LasA activity. LasA did not induce JAZ1-GUS degradation in the complete loss-of-function *coi1-1* mutant (Fig. 4A, B) and, as expected, JA also did not promote JAZ1-GUS degradation in this mutant. The JA-Ile analogue COR acts as a ligand of the COI1-JAZ receptor and activates JAZ degradation and JA responses independently of JAR1 (Sheard *et al.*, 2010) and indeed, our results showed that COR induced JAZ1 degradation in a COI1-dependent, JAR1-independent manner (Fig. 4A). Finally, LasA did not prevent hormone-triggered degradation of JAZ1 during concurrent JA and LasA treatment (Supplementary Fig. S3).

These results show that LasA induces JAZ degradation, and that its activity requires JAR1-mediated JA conversion into JA-Ile, as well as COI1-dependent perception of JA-Ile.

## Discussion

Jasmonates are signalling molecules that govern plant responses to stress. Jasmonic acid is the direct precursor of the bioactive plant hormone JA-Ile, the endogenous ligand of the plant receptor complex. Several fungi produce JA and JA derivates; phytotoxic activity is reported for most of these compounds, although a precise molecular mode of action has yet to be proposed. Here, we addressed the activity of the natural JA derivate LasA, which is produced by the pathogenic fungus *Lasiodiplodia mediterranea* (Andolfi *et al.*, 2014). Our results showed that LasA activated the plant JA pathway and that its activity required JAR1 and COI1, key proteins required for JA conversion into JA-Ile and for JA-Ile perception, respectively. Because LasA is a fungal JA furanonyl ester, we propose that this naturally occurring fungal compound can be catabolized to release JA, which is in turn converted into JA-Ile by JAR1. In support of this hypothesis, LasA activity required JAR1 for JA conjugation to Ile, as well as recognition by the JA-Ile perception complex. LasA might therefore function as an inactive JA conjugate that can be hydrolysed to liberate the direct JA-Ile precursor JA.

A cleaving amidohydrolase activity on JA conjugates has been described for the JA-producing fungus *Botryodiplodia theobromae* (Hertel *et al.*, 1997). This enzymatic activity might be conserved among fungi, which would explain the detection of notable amounts of JA in several species with only minimal accumulation of JA-conjugated molecules (Chanclud and Morel, 2016; Lo Presti *et al.*, 2015; Eng *et al.*, 2016; Fonseca *et al.*, 2018); however, the fungal cleaving amidohydrolase activity on JA conjugates might mask identification of additional natural JA-conjugates under *in vitro* conditions. Plants have also evolved amidohydrolase activity; the plant enzyme IAR3/ILL6 deconjugates JA-Ile into JA (Woldemariam *et al.*, 2012; Bhosale *et al.*, 2013; Widemann *et al.*, 2013). This process might accept different substrates, since IAR3/ILL6 can also deconjugate 12OH–JA-Ile into 12OH-JA (Widemann *et al.*, 2013). In addition, several members of this amidohydrolase family release free auxin (IAA) by cleaving IAA–amino acid conjugates, with each hydrolase deconjugating a different subset of the conjugates (LeClere *et al.*, 2002). Finally, the expression of several amidohydrolase genes is induced by auxins and jasmonate, strengthening the case for the involvement of amidohydrolase in hormone signalling (LeClere *et al.*, 2002; Zhang *et al.*, 2016). Cleaving amidohydrolase activity of molecule–amino acid conjugates is thus a fairly common mechanism in nature, and both fungi and plants might be able to hydrolyse JA-conjugates such as LasA.

LasA activation of JA responses is dependent on the JA-Ile co-receptor COI1. A similar requirement for the plant COI1 JA-Ile receptor was reported for the virulent activity of fungal JA-conjugates produced by *Fusarium oxysporum* (Miersch *et al.*, 1999; Brodhun *et al.*, 2013; Cole *et al.*, 2014). These data suggest that different fungi have evolved distinct JA-conjugates with similar biological activity dependent on the plant COI1 JA-Ile receptor.

Jasmonic acid regulates several plant responses, including plant defences against necrotroph pathogens, fungi, and herbivores (Wasternack and Hause, 2013; Goossens *et al.*, 2016; Guo *et al.*, 2018). On the other hand, activation of the JA pathway weakens the defences against biotrophic and hemibiotrophic pathogens triggered by salicylic acid (SA) (Robert-Seilaniantz *et al.*, 2011). Hence, some strains of *Pseudomonas syringae* produce a mimic of the bioactive JA-Ile hormone, coronatine, to activate the JA pathway, which in turn inhibits the SA-dependent defences required for *P. syringae* resistance (Cui *et al.*, 2005; Robert-Seilaniantz *et al.*, 2011). A biotrophic or hemibiotrophic infection stage for *L. mediterranea* has not been reported to date, providing a case against the hypothesis that *L. mediterranea* produces LasA to activate JA-dependent responses to inhibit SA defences.

The suggested mode of action for fungal LasA—that it functions as a pool of inactive conjugated JA that is converted to active JA, and hence activates plant JA responses including defence against necrotrophic fungi—might seem counterintuitive at first glance. However, necrotrophic fungi induce plant cell death, which is beneficial for fungal growth and proliferation (Chanclud and Morel, 2016; Lo Presti *et al.*, 2015). We therefore propose that fungal production of LasA, and possibly of additional JA derivates, would be spatio-temporally regulated to activate JA-mediated senescence and cell death only in specific conditions. For example, to avoid inducing plant defences, fungi would not produce LasA in early infection stages, whereas production and subsequent induction of plant cell death would occur in late stages in order to facilitate fungal propagation and infection. Future analysis of *Lasiodiplodia* mutants with impaired LasA production will test this hypothesis.

## Supplementary data

Supplementary data are available at *JXB* online.

Fig. S1. Accumulation of anthocyanins in response to exogenous treatment with JA, LasA, or COR.

Fig. S2. Effects of LasA on JA-regulated transcriptional activation.

Fig. S3. Effect of LasA on JA-mediated JAZ degradation.

Table S1. Data for root growth measurements reported in Fig. 1F.

Supplementary Figures and Tables
