# Supplementary material for: The fungal phytotoxin lasiojasmonate A activates the plant jasmonic acid pathway
Source: J Exp Bot. 2018 Mar 24;69(12):3095–102. doi: 10.1093/jxb/ery114 (PMC5972634; doi:10.1093/jxb/ery114)
Supplement: Supplementary Figures and Tables [file ery114_suppl_supplementary_figures_tables.pdf]

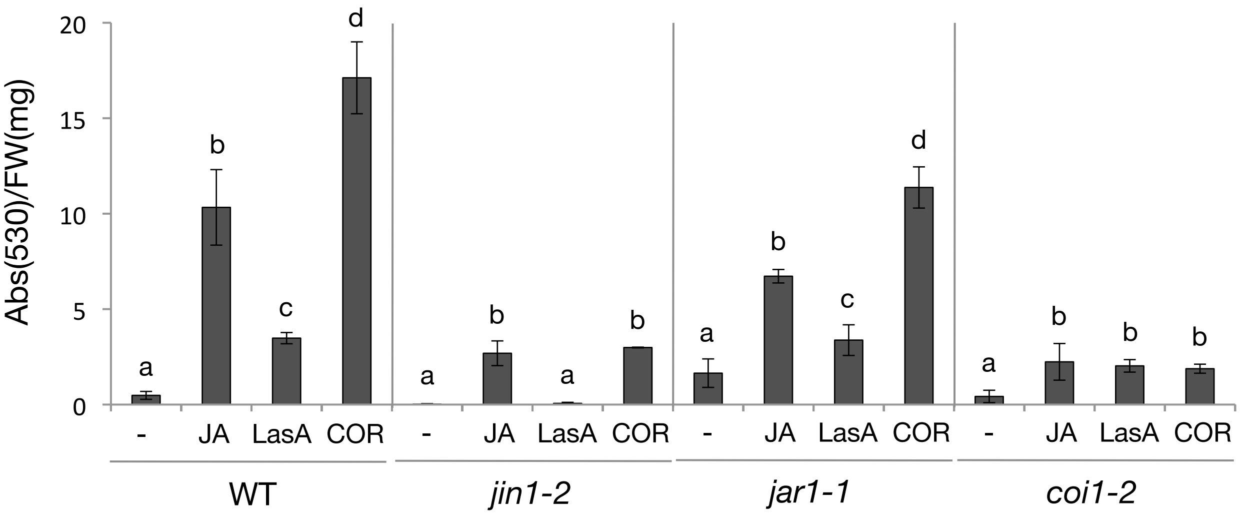

Supplementary Figure 1. Accumulation of anthocyanins in response to exogenous treatment with JA, LasA or COR.

Wild-type (Col-0) mutant seedlings (N = 15-25) were germinated in absence (-) or presence of 50 μM JA, 50 μM LasA or 0.5 μM COR. Ten days after germination, anthocyanin accumulation [Abs(530nm)/fresh weight (mg)] was measured. Data show mean ± SD. Letters stand for statistical differences (One-way ANOVA with post-hoc Tukey HSD, $p<0.05$) compared to the untreated control per each plant genotype.

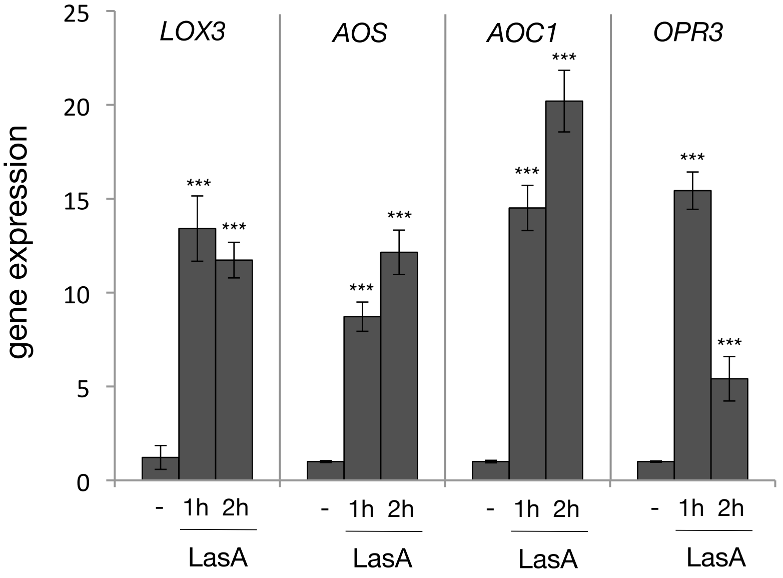

Supplementary Figure 2. Effect of LasA on JA-regulated transcriptional activation
Gene expression analysis of the JA biosynthetic genes *LOX3*, *AOS*, *AOC5* and *OPR3* in wild-type (Col-0) plants in response to 50 µM LasA for 1 or 2 hours; untreated plants (-) are included as control. *ACT8* was used as housekeeping control gene. Statistically significant gene expression differences between control and LasA-treated plants are identified with asterisks (Two-tailed Student's t-test; *** $P < 0.001$). Each biological sample consisted of tissue pooled from 10-15 plants. Data show mean ± SD of four technical replicates.

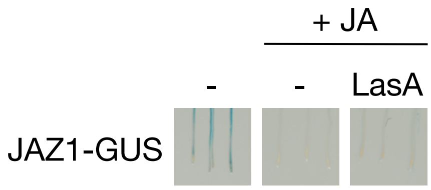

Supplementary Figure 3. Effect of LasA on JA-mediated JAZ1 degradation
GUS visualization of JAZ1-GUS in root tips of 7-day-old transgenic JAZ1-GUS Arabidopsis. 35S:JAZ1-GUS wild-type plants (N = 20) were treated with 5 μM JA alone or concurrently with 100 μM of LasA for 1h. Untreated control plants are shown as (-).

Supplementary Table 1. Data of root growth measurements reported in Figure 1F.

The fungal phytotoxin lasiojasmonate A activates the plant jasmonic acid pathway

treatment	control	JA	LasA	COR	control	JA	LasA	COR	control	JA	LasA	COR	control	JA	LasA	COR	control	JA	LasA	COR	treament
plant genotype	Col-0	Col-0	Col-0	Col-0	jin1-2	jin1-2	jin1-2	jin1-2	ja3-1	ja3-1	ja3-1	ja3-1	jar1-1	jar1-1	jar1-1	jar1-1	coi1-2	coi1-2	coi1-2	coi1-2	plant genotype
Average	33.82	6.962	6.401	6.019	33.46	19.9	17.34	18.97	33.5	20.01	15.47	15.3	33.07	19.75	24.15	6.05	33.09	25.34	25.82	22.39	Average
Standard Dev	4.695	0.7	0.789	1.258	3.106	3.826	1.453	2.323	4.574	5.741	2.763	3.294	4.03	3.391	2.559	0.849	1.836	6.225	2.877	2.342	Standard Dev
ANOVA vs CTL		**	**	**		**	**	**		**	**	**		**	**	**		**	**	**	ANOVA vs CTL
ANOVA vs JA	**		ns	ns	**		ns	ns	**		ns	ns	**		ns	**	**		ns	ns	ANOVA vs JA
